# Dietary L-Lysine Requirement of Coho Salmon (*Oncorhynchus kisutch*) Alevins

**DOI:** 10.3390/ani13233670

**Published:** 2023-11-27

**Authors:** Leyong Yu, Hairui Yu, Ziyi Yuan, Jiayi Zhang, Lingyao Li, Chengyu Ma, Weiguang Kong

**Affiliations:** 1 Weifang Key Laboratory of Coho Salmon Culturing Facility Engineering, Institute of Modern Facility Fisheries, College of Biology and Oceanography, Weifang University, Weifang 261061, China; leyong618@gmail.com (L.Y.); moonn828@163.com (Z.Y.); zhangjiayiazan1005@163.com (J.Z.); 980714742@163.com (L.L.); mcy3199268616@163.com (C.M.); 2 Shandong Collaborative Innovation Center of Coho Salmon Health Culture Engineering Technology, Shandong Conqueren Marine Technology Co., Ltd., Weifang 261108, China; 3 Department of Hydrobiology, College of Fisheries and Life Science, Shanghai Ocean University, Shanghai 201306, China; 4 Key Laboratory of Breeding Biotechnology and Sustainable Aquaculture, Institute of Hydrobiology, Chinese Academy of Sciences, Wuhan 430072, China

**Keywords:** essential amino acid, L-lysine, *Oncorhynchus kisutch* alevins, protein efficiency ratio, feed efficiency

## Abstract

**Simple Summary:**

With the increasing demand for aquaculture, the resource of high-quality traditional protein (such as fishmeal) becomes limited, and plant ingredients are often used as substitutes for fishmeal because of their low price and wide availability. However, plant proteins are usually deficient in certain essential amino acids (EAAs), which adversely affects the growth performance of aquatic animals. Lysine is generally the first limiting amino acid in plant protein ingredients used to formulate the aquafeed, and it plays an important role in physiological processes including antioxidant, differentiation, growth, immunity, and reduction of nitrogen emission. In this study, the effects of dietary graded L-lysine concentrations on the growth, proximate composition, and AA (amino acid) profile of coho salmon alevins were evaluated by a feeding trial. The results showed that an adequate dietary lysine level significantly improved the specific growth rate (SGR), protein efficiency ratio (PER), body protein deposition (BPD), and feed conversion ratio (FCR). The dietary optimum L-lysine requirements for coho salmon alevins were 3.74%, 3.73%, 3.91%, and 3.77% of the diet or 6.80%, 6.78%, 7.11%, and 6.85% of dietary proteins against the SGR, PER, BPD, and FCR, respectively.

**Abstract:**

The suitable dietary L-lysine concentration for coho salmon (*Oncorhynchus kisutch*) alevins was assessed by a dose response feeding trial. Six experimental diets were made with graded L-lysine concentrations of 2.29%, 2.81%, 3.32%, 3.80%, 4.27%, and 4.78% of the dry matter, respectively, each of which was fed to triplicate groups of 100 alevins (initial body weight: 0.30 ± 0.01 g) in 18 plastic baskets (water volume 240 L). The alevins were cultured in a flowing freshwater system and fed manually to apparent satiation four times a day for 12 weeks. The survival rate of alevins did not differ significantly among the dietary groups. The specific growth rate (SGR), protein efficiency ratio (PER), and body protein deposition (BPD) increased significantly (*p* < 0.05) with the increase in dietary lysine concentration up to 3.80% and then reduced as lysine level further increased. The feed conversion ratio (FCR) had an inverse trend to SGR. The whole-body crude protein content of the alevins increased significantly with increasing dietary lysine level, while crude lipid content showed the opposite trend. In comparison, the contents of morphological indices, whole-body moisture, and ash were not affected significantly (*p* > 0.05) by the different dietary lysine concentrations. The highest contents of lysine, arginine, and total essential amino acids (EAAs) were observed in the group with 4.27% dietary lysine concentration, which did not differ significantly from those in the 3.32%, 3.80%, and 4.78% groups but was significantly higher than those in the 2.29% and 2.81% groups. Similarly, valine had the highest content in the group with 4.78%. The variations in dietary lysine had no significant impacts on other EAA and non-EAA contents except glycine, which increased with increasing dietary lysine level. Second-order polynomial model analyses based on SGR, PER, BPD, and FCR evaluated the optimum L-lysine requirements of coho salmon alevins as 3.74%, 3.73%, 3.91%, and 3.77% of the diet or 6.80%, 6.78%, 7.11%, and 6.85% of dietary proteins, respectively.

## 1. Introduction

Fishmeal (FM) is often used as the preferred source of animal protein for aquafeed owing to its ideal digestible protein ratio, well-balanced essential amino acid (EAA) structure, and low anti-nutritional factors [1,2,3,4]. However, with the rapid development of the aquaculture and aquafeed industry, the supply and demand of FM are unbalanced due to the unsustainability of fishery resources, rising cost, and shortage of FM supply [5,6,7]. Therefore, exploring alternatives to FM has become a hot topic of research in the past several decades. Plant proteins including soybean meal [8,9], peanut meal [10,11], rapeseed meal [12,13], and cottonseed meal [14,15] have been widely researched as substitutes for FM in aquafeed. However, plant proteins are usually deficient in some EAAs, which need to be taken into consideration when formulating feeds with plant proteins [16,17]. Thus, optimizing the dietary amino acid supplementation is very important for specific fish species.

The most restrictive EAAs include lysine, methionine, and threonine, of which lysine is generally the first limiting amino acid in plant protein ingredients used to prepare aquafeed [18,19,20,21]. As an exogenous amino acid, lysine plays an important role in physiological processes including antioxidant, differentiation, growth, immunity, and reduction of nitrogen emission [22,23,24]. Lysine is taken up by intestinal villus cells in the form of L-lysine and enters directly into tissues and organs, where it is involved in protein synthesis and deposition and weight gain and body length in fish, and without it, other amino acids are restricted to be used [24,25].

Previous studies have shown that lysine deficiency leads to poor growth and low feed utilization for fish [26,27]. Meanwhile, lysine is associated with the fish immune system since adequate lysine level can modulate the immune response [22,28]. The nutrient requirement varied with different stages of growth, whereas the L-lysine requirement decreased gradually with live body weight increase in Atlantic salmon (*Salmo salar*) and rainbow trout (*Oncorhynchus mykiss*) [18]. Additionally, the ratio of endogenous amino acid loss and growth requirement varied in different growth stages [29]. Thus, it is necessary to determine the optimal dietary lysine levels in fish at different stages.

Coho salmon (*Oncorhynchus kisutch*), one species of Pacific salmon, is widely dispersed throughout its natural ranges, from the Bering Sea in the Russian Far East to Alaska and along the coast of South America to California, and is of great significance to commercial facility fisheries [30]. Coho salmon has become an increasingly popularly cultured salmonid species in China due to it being rich in beneficial highly unsaturated fatty acids and other functional health-care substances [31,32]. However, with the expansion of aquaculture and the increase in feed demand, there is still scare understanding of the nutrition including the dietary lysine requirement for coho salmon. Therefore, the effects of dietary L-lysine supplementation on the growth, proximate composition, and AA profile of coho salmon alevins were studied to estimate the suitable L-lysine concentration in diet.

## 2. Materials and Methods

### 2.1. Experimental Diets

The amino acid profile of coho salmon eggs was analyzed in order to formulate the experimental diets. The coho salmon eggs in eyed stage were provided by Conqueren Leading Fresh Science & Technology Inc., Ltd. (Weifang, China), and the samples were stored at −20 °C and then freeze-dried to determine the proximate composition and amino acid profile (Table 1).

The formulation, proximate composition, and amino acid profile of the experimental diets and amino acid profile of the ingredients are shown in Table 2 and Table 3, respectively. The lysine concentration in the basal diet was the lowest, accounting for 2.29% of the dry matter or 4.13% of the dietary protein, which came from FM, poultry by-product meal, soy protein concentrate, corn gluten meal, wheat gluten meal, and beer yeast. The five other diets contained graded L-lysine concentrations (2.81%, 3.32%, 3.80%, 4.27%, and 4.78% of the dry matter or 5.11%, 6.04%, 6.91%, 7.76%, and 8.69% of the dietary protein, respectively) by adding different concentrations of L-lysine hydrochloride (Juneng Golden Corn Development Co., Ltd., Weifang, China). In addition to lysine, crystal amino acids were supplemented to maintain the dietary EAA composition consistent with those of coho salmon eyed eggs. When the lysine concentration was increased, the experimental diet was kept isonitrogenous (8.8% nitrogen) and isoenergetic (21.57 kJ/g gross energy) by reducing the corresponding content of glycine. The dietary pH was adjusted to 7.0–7.5 with 6 mol/L NaOH solution [33]. All the diets were made by low temperature extrusion, then air-dried and packed in separate bags and stored at −20 °C until used.

### 2.2. Animals and Experimental Protocols

All animal experimental protocols were approved by the Regulations of Weifang University on the Management of Experimental Animal (approval number 2022032606) and were in compliance with the Regulations of Shandong Province on the Management of Experimental Animals. The broodfish of coho salmon, each with a mean body weight of about 4.5 kg, were selected for providing the fertilized eggs. After the fertilized eggs developed to the eyed stage, the same batch of eyed eggs was analyzed for amino acid profile as mentioned above, and they were incubated at one base of Conqueren Leading Fresh Marine Science & Technology Inc., Ltd. (Weifang, China). The alevins hatched out were gradually conditioned to the rearing environment and fed the basal diet. After fasting for 24 h, 100 alevins (individual body weight: 0.30 ± 0.01 g) were assigned to 18 plastic baskets (80 × 60 × 60 cm, water capacity 240 L) with three baskets per diet. All the baskets were placed in an indoor culture pond (700 cm × 500 cm × 150 cm). The alevins were cultured in filtered freshwater (the dissolved oxygen, 9.5 ± 0.8 mg/L; pH value, 6.9 ± 0.3; water temperature, 15.5 ± 0.5 °C) with a natural light–dark cycle and fed manually to apparent satiation four times (6:45, 10:15, 13:45, 17:15) a day for 12 weeks.

### 2.3. Sampling Procedures

One hundred fish at the start of the feeding trial were sampled for proximate composition analysis. After fasting for 24 h, all the alevins in each basket were counted and weighed. Thirty alevins in each basket were sampled and stored at −20 °C for determining the final whole-body proximate composition and amino acid profile. Other twenty alevins anesthetized with a concentration of 20 mg/L of tricaine methane sulfonate (MS-222) were sampled for determining the morphological indicators (condition factor, CF; hepatosomatic index, HSI; viscerosomatic index, VSI).

### 2.4. Analytical Methods

Proximate composition of the ingredients, diets, and alevin samples was analyzed according to AOAC [34]. Briefly, the dry matter content was measured by drying to a constant weight at 105 °C. Crude protein, crude lipid, and ash contents were measured by determining nitrogen (N × 6.25) using the Kjeldahl method, ether extraction using the Soxhlet method, and by muffle furnace heating at 550 °C for 24 h, respectively. The amino acid (except tryptophan) contents were analyzed by an automatic amino acid analyzer (Model A300, MembraPure GmbH, Frankfurt, Germany). The samples were freeze-dried and hydrolyzed with 6 mol/L HCl solution at 110 °C for 24 h, then the hydrolysate was filtered, vacuum-dried 2 mL of the hydrolysate after constant volume was taken, and 2 mL of 0.02 mol/L HCl solution was added, which was vibrated constantly to dissolve all the amino acids and then chromatography was performed. Total energy was analyzed with a Parr 1281 automated oxygen bombardment meter (Parr, Moline, IL, USA).

### 2.5. Calculation Methods

The following formulae were used to calculate relative parameters:

Survival rate (%) = 100 × N_f_/N_i_ (1)

Specific growth rate (SGR, %/day) = 100 × (ln (BW_f_ (g)) − ln (BW_i_ (g)))/d (2)

Feed conversion ratio (FCR) = FI (g)/(BW_f_ (g) − BW_i_ (g)) (3)

Protein efficiency ratio (PER, %) = (BW_f_ (g) − BW_i_ (g))/(FI (g) × FP (%)) (4)

Body protein deposition (BPD, %) = 100 × (BWf (g) × CP_f_ (%) − BWi (g) × CP_i_ (%))/(FI (g) × FP (%)) (5)

CF (g/(cm)^3^) = 100 × BW_f_ (g)/(BL_f_ (cm)^3^) (6)

HSI (%) = 100 × LW_f_ (g)/BW_f_ (g) (7)

VSI (%) = 100 × VW_f_ (g)/BW_f_ (g) (8)

where N_f_ and N_i_, BW_f_ and BW_i_, and CP_f_ and Cp_i_ were the final and initial numbers, final and initial body weight, and final and initial carcass protein content of fish, respectively; FI and FP were the feed intake and feed protein content, respectively; BL_f_, LW_f_, and VW_f_ were the final body length, final liver weight, and final visceral mass weight, respectively; and d was the feeding period in days.

### 2.6. Statistical Analysis

The data were presented as mean ± standard deviation (mean ± S.D.) and all the statistical analyses were performed using SPSS 25.0 software (Chicago, IL, USA). All percentage data were converted to inverse sine prior to analysis. Second order regression analysis was used to quadratically analyze optimum L-lysine levels in diet based on SGR, PER, BPD, and FCR, respectively [35]. A significance level was set at *p* < 0.05.

## 3. Results

### 3.1. Survival and Growth Performance

**Figure 1:**
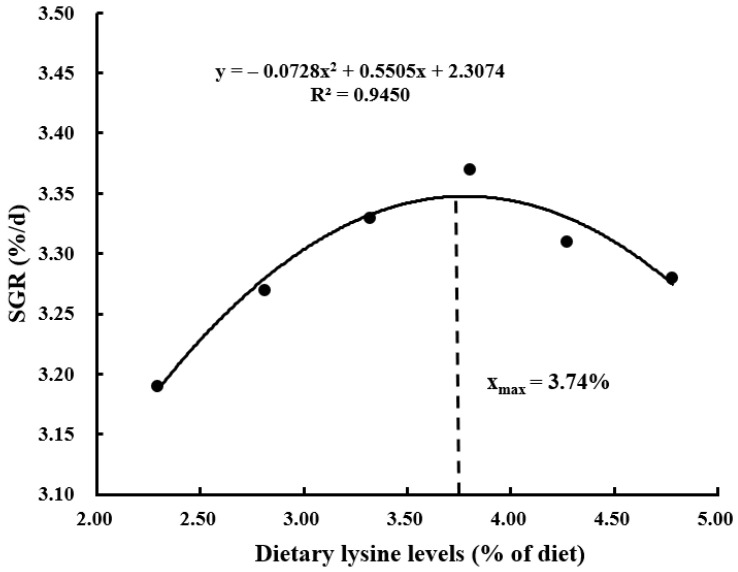
A second-order polynomial analysis based on specific growth rate (SGR) showed that the optimum lysine requirement was 3.74% of the diet or 6.80% of dietary proteins for coho salmon (*Oncorhynchus kisutch*) alevins.

**Figure 2:**
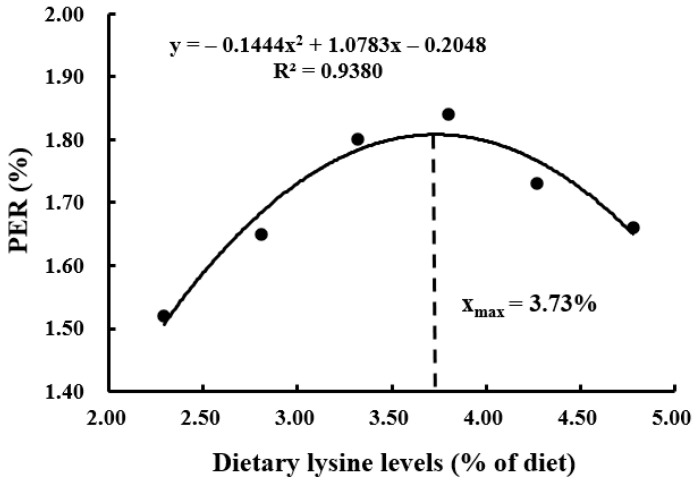
A second-order polynomial analysis based on protein efficiency ratio (PER) showed that the optimum lysine requirement was 3.73% of the diet or 6.78% of dietary proteins for coho salmon (*Oncorhynchus kisutch*) alevins.

**Figure 3:**
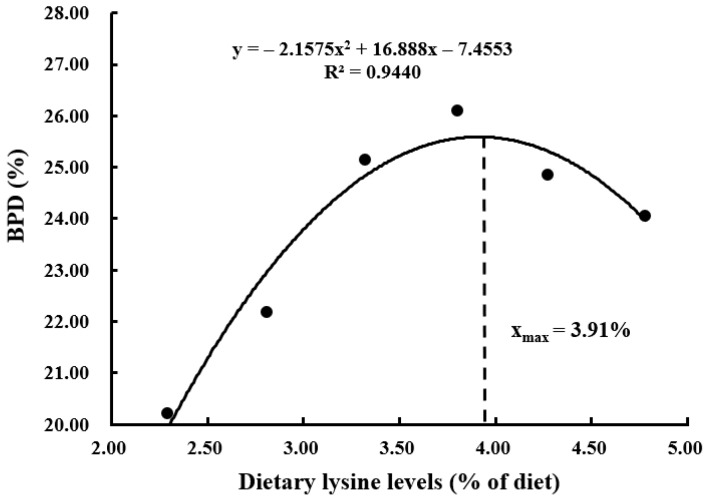
A second-order polynomial analysis based on body protein deposition (BPD) showed that the optimum lysine requirement was 3.91% of the diet or 7.11% of dietary proteins for coho salmon (*Oncorhynchus kisutch*) alevins.

**Figure 4:**
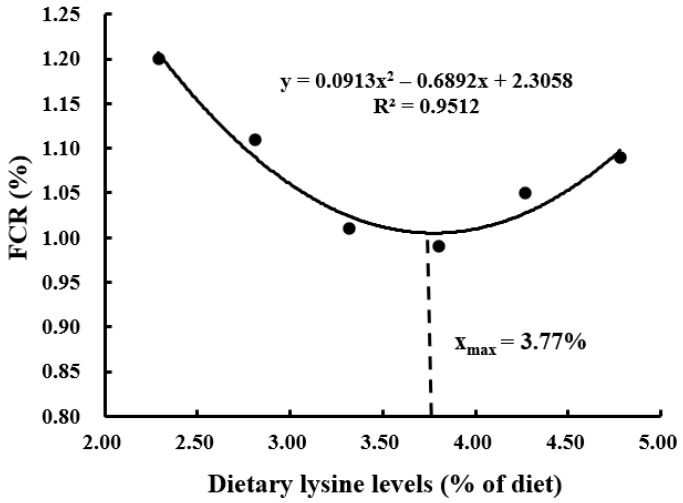
A second-order polynomial analysis based on feed conversion ratio (FCR) showed that the optimum lysine requirement was 3.77% of the diet or 6.85% of dietary proteins for coho salmon (*Oncorhynchus kisutch*) alevins.

Survival rates of the alevins were not affected significantly (*p* > 0.05) by the variations in dietary L-lysine (Table 4). The SGR, PER, BPD, and FCR parameters of the alevins were affected significantly (*p* < 0.05) by different dietary L-lysine levels. The SGR increased significantly (*p* > 0.05) with increasing L-lysine level in the diet up to 3.80% and then reduced as the lysine level further increased. A second-order polynomial model analysis based on SGR and dietary L-lysine concentrations showed that the optimum L-lysine requirement for coho salmon alevins was estimated at 3.74% of the diet or 6.80% of dietary proteins (Figure 1, *y* = −0.0728x^2^ + 0.5505x + 2.3074, R^2^ = 0.9450). PER and BPD showed the same trends as SGR, and the optimum L-lysine requirements were 3.73% of the diet or 6.78% of dietary proteins (Figure 2, *y* = −0.1444x^2^ + 1.0783x − 0.2048, R^2^ = 0.9380) and 3.91% of the diet or 7.11% of dietary proteins (Figure 3, *y* = −2.1575x^2^ + 16.888x − 7.4553, R^2^ = 0.9440), respectively. An opposite trend was found for FCR, which was the lowest in the group with 3.80%, and the optimum L-lysine requirement was 3.77% of the diet or 6.85% of dietary proteins (Figure 4, y = −0.0913x^2^ + 0.6892x + 2.3058, R^2^ = 0.9512). The group with 3.32% lysine level had significantly higher feed intake than the groups with 2.28%, 2.81%, and 4.78%. The different concentrations of dietary L-lysine had no significant effects on the CF, HSI, and VSI values.

### 3.2. Proximate Whole-Body Composition

The whole-body crude protein content ranged from 13.25% to 14.33%, which increased significantly (*p* < 0.05) with increasing dietary lysine concentration (Table 5). The crude lipid content was the highest in the group with 2.29% dietary lysine and ranged from 4.96% to 5.57%, showing the opposite trend. In contrast, the variation in dietary L-lysine concentrations had no significant (*p* > 0.05) effects on the contents of moisture and ash, which ranged 77.48–78.02% and 3.98–4.20%, respectively.

### 3.3. Whole-Body EAA Profile

The highest contents of lysine, arginine, and total EAAs were observed in the group with 4.27% dietary lysine concentration (Table 6), which did not differ significantly (*p* > 0.05) from those in the 3.32%, 3.80%, and 4.78% groups but was significantly (*p* < 0.05) higher than those in the 2.29% and 2.81% groups. Similarly, valine exhibited the highest content in the group with 4.78%. The change in dietary lysine had no significant effect on other EAAs and NEAAs except glycine, which was increased with increasing dietary lysine level.

## 4. Discussion

Lysine is an EAA for the cultured fish species so far investigated [18], which is associated with the nutrition metabolism and growth [36]. Previous studies showed that lysine deficiency might lead to a loss of appetite, causing reduced dietary intake, feed utilization, and growth rate [20,37]. The SGR, PER, BPD, and FCR of the alevins were poor with inadequate dietary lysine content and were significantly improved with the suitable dietary lysine concentration, with the SGR, PER, and BPD exhibiting an increasing trend followed by a decreasing trend while the FCR displayed the opposite trend. Similar results were observed for juvenile turbot (*Psetta maxima*) [38] and rainbow trout (*Salmo gairdneri*) [39,40]. A lysine-deficient diet had led to high mortality and caudal fin erosion rates in rainbow trout [38], while excessive dietary lysine could also negatively affect the growth performance [27,37]. In comparison, several studies reported that lysine supplementation in diet had no significant effects on the growth rates in rainbow trout [39], *Colossoma macropomum* (Cuvier, 1818) [24], and grass carp (*Ctenopharyngodon idella*) [36]. In the present study, the optimum dietary lysine requirements were estimated at 6.78%-7.11% of dietary proteins for coho salmon alevins, which were lower than the requirements obtained for black sea bream (*Sparus macrocephalus*) at 8.64% [41] but higher than the requirements for catla (*Catla catla*) at 6.20% [42], rainbow trout at 6.10% [43], Japanese seabass (*Lateolabrax japonicus*) at 5.80% [37], Atlantic salmon (*Salmo salar*) at 5.37% [44], cobia (*Rachycentron canadum*) at 5.30% [45], and Pacific threadfin (*Polydactylus sexfilis*) at 5.10% [46]. Many studies have demonstrated that the growth and health status may depend on many factors such as feeding period, water quality, fish size, as well as the dietary nutrient concentrations [47], which might explain the higher L-lysine requirement obtained for coho salmon alevins. Moreover, compared with the larger fish, the early stage of fish has a higher potential rate of growth and requires higher amounts of amino acids to meet their higher protein synthesis, energy metabolism, and other special physiological functions [48,49].

The values of CF, HSI, and VSI in the alevins were not significantly affected by the different concentrations of dietary lysine. Similarly, the dietary lysine supplementation had no significant influence on the HSI of *Megalobrama amblycephala* [50]. The intestosomatic index reduced with increasing the dietary lysine up to 1.29% and then slightly increased, while the VSI value was significantly lower in groups with 0.85% and 1.29% lysine levels than in the group with 1.50% lysine level in the diet for grass carp [36]. To a great extent, the morphological parameters were mainly influenced by dietary macro-nutrients such as proteins, lipids, and carbohydrates [51].

Lysine, one of the ketogenic amino acids, is involved in energy metabolism and affects the body composition [27,38,52]. When the body is low in carbohydrates, lysine can be broken down into glucose or ketone bodies to provide energy. Together with methionine, lysine acts as a precursor of carnitine involved in the transfer of long-chain fatty acyl groups into mitochondria for beta-oxidation and plays an important physiological role in lipid metabolism [39]. Helland and Grisdale-Helland [52] found that changes in dietary lysine had a significant effect on the proximate composition of Atlantic halibut (*Hippoglossus hippoglossus*) with the lipid deposition decreasing with increasing dietary lysine level. Supplementation of lysine and carnitine, one or both, to the diet could significantly reduce the whole-body lipid content and increase the protein content [53,54]. Dietary lysine supplementation had also promoted the protein synthesis and deposition in *Megalobrama amblycephala* [50], *Psetta maxima* [55], and *Scophthalmus maximus* [56]. These results reveal that body composition changes are a parameter reflecting the level of amino acid availability besides the evaluation indicators of weight gain and feeding efficiency. Similar results are shown in the current study that different lysine levels significantly influenced the protein and lipid contents, in which the crude protein content increased while the crude lipid content decreased with the increase in dietary lysine level. This variation might be due to the effect of L-lysine deficiency in the feed on carnitine synthesis, resulting in reduced decomposition and abnormal deposition of lipid, further increasing the body lipid content of the coho salmon alevins, which is consistent with the findings in channel catfish (*Ictalurus punctatus*) [53].

In general, the distribution of amino acids in a specific protein is fixed, whereas the whole-body protein distribution in fish and terrestrial animals varies with age and growth [37,57]. Rodehutscord et al. [58] found that dietary lysine concentration affected the amino acid composition of rainbow trout, which was consistent with the findings of coho salmon alevins. Moreover, lysine supplementation did not only affect the growth and protein retention but also affected the body lysine concentration of coho salmon alevins. Similar results have also been found in rainbow trout [58,59] and large yellow croaker (*Pseudosciaena crocea* R.) [27]. Different composition and ratios of amino acids in the diet have an impact on the distribution of amino acids in the coho salmon alevin tissues. The body lysine levels increased with increasing dietary lysine levels, then tended to stabilize, which was possibly due to the “protein leverage” effect, whereby the total energy intake decreases with the increase in dietary protein level to maintain a constant absolute protein intake, and vice versa [39,60,61]. Meanwhile, Kroeckel et al. [38] found that dietary lysine concentrations affected the contents of lysine, methionine, histidine, isoleucine, leucine, and phenylalanine in the whole-body protein of juvenile turbot, which was different from the results in this study, in which other EAAs were unaffected by dietary L-lysine concentrations, apart from lysine, valine, and arginine. Differences in dietary lysine intake by different species, or even the same species of fish, may be related to the fish size and age, feeding regime, other nutrient levels, population density, water temperature, flowing rate, as well as other experimental conditions [25].

## 5. Conclusions

This research demonstrated that adequate dietary lysine level could significantly improve the SGR, PER, BPD, and FCR of coho salmon alevins, affecting the contents of crude protein, lipid, lysine, arginine, valine, and total EAAs. Second-order polynomial model analyses based on SGR, PER, BPD, and FCR showed that the optimum L-lysine requirements of coho salmon alevins were 3.74%, 3.73%, 3.91%, and 3.77% of the diet or 6.80%, 6.78%, 7.11%, and 6.85% of dietary proteins, respectively. The data would be helpful in the development of lysine-balanced feed for coho salmon. Future research could be extended to determine the digestibility of protein ingredients for this farmed fish species.

## Figures and Tables

**Table 1:** Proximate composition and amino acid profile of coho salmon (*Oncorhynchus kisutch*) eggs in eyed stage (% dry matter) ^1^.

Composition	Coho Salmon Eggs in Eyed Stage	Percentage of Amino Acid in Relation to Lysine
Proximate composition		
Crude protein	55.78 ± 0.39	
Crude lipid	18.97 ± 0.46	
Ash	5.74 ± 0.41	
Essential amino acid (EAA) profile ^2^		
Arginine	3.18 ± 0.06	78.33 ± 0.52
Histidine	1.59 ± 0.04	39.16 ± 0.56
Isoleucine	3.07 ± 0.06	75.62 ± 0.65
Leucine	4.83 ± 0.08	118.97 ± 0.72
Lysine	4.06 ± 0.06	
Methionine	2.28 ± 0.03	56.16 ± 0.35
Phenylalanine	2.78 ± 0.03	68.47 ± 0.29
Threonine	2.70 ± 0.05	66.50 ± 0.51
Valine	3.82 ± 0.04	94.09 ± 0.43
Non-essential amino acid (NEAA) profile		
Alanine	4.11 ± 0.05	101.23 ± 0.53
Aspartic acid	4.94 ± 0.04	121.67 ± 0.49
Glutamic acid	6.38 ± 0.07	157.14 ± 0.86
Glycine	2.14 ± 0.02	52.71 ± 0.36
Proline	2.78 ± 0.02	68.48 ± 0.29
Serine	3.47 ± 0.05	85.47 ± 0.67
Tyrosine	1.99 ± 0.02	49.01 ± 0.47
Cysteine	0.49 ± 0.01	12.07 ± 0.07

^1^ Values are shown as mean ± S.D. (standard deviation, *n* = 3). ^2^ Tryptophan was not determined due to acid hydrolysis.

**Table 2:** Formulation, proximate composition, and amino acid profile of the experimental diets (% dry matter).

Dietary Groups	Analyzed Dietary L-Lysine Level (% of Diet)					
	2.29	2.81	3.32	3.80	4.27	4.78
Fish meal (Peruvian) ^1^	15.00	15.00	15.00	15.00	15.00	15.00
Poultry by-product meal ^1^	10.00	10.00	10.00	10.00	10.00	10.00
Soy protein concentrate ^1^	15.00	15.00	15.00	15.00	15.00	15.00
Corn gluten meal ^1^	10.00	10.00	10.00	10.00	10.00	10.00
Wheat gluten meal ^1^	10.00	10.00	10.00	10.00	10.00	10.00
Beer yeast ^1^	5.00	5.00	5.00	5.00	5.00	5.00
L-lysine.HCl	0.00	0.64	1.28	1.92	2.56	3.20
Glycine ^1^	2.55	2.04	1.53	1.02	0.51	0.00
Amino acid mixture ^2^	7.59	7.59	7.59	7.59	7.59	7.59
Cellulose	0.65	0.52	0.39	0.26	0.13	0.00
α-starch	10.78	10.78	10.78	10.78	10.78	10.78
Fish oil	5.00	5.00	5.00	5.00	5.00	5.00
Soybean oil	5.00	5.00	5.00	5.00	5.00	5.00
Soy lecithin	1.00	1.00	1.00	1.00	1.00	1.00
Monocalcium phosphate	1.00	1.00	1.00	1.00	1.00	1.00
Vitamin premix ^3^	0.50	0.50	0.50	0.50	0.50	0.50
Mineral premix ^4^	0.50	0.50	0.50	0.50	0.50	0.50
Choline chloride	0.30	0.30	0.30	0.30	0.30	0.30
Ascorbyl polyphosphate	0.10	0.10	0.10	0.10	0.10	0.10
Antioxidant	0.03	0.03	0.03	0.03	0.03	0.03
Proximate composition (*n* = 3)						
Crude protein	54.81	54.88	55.06	55.02	55.05	55.14
Crude lipid	15.33	15.35	15.29	15.25	15.31	15.47
Ash	8.21	8.26	8.34	8.37	8.29	8.38
Gross energy (KJ/g DM)	21.55	21.61	21.52	21.46	21.67	21.63
Amino acid profile (% of diet)						
Essential amino acid (EAA) profile						
Lysine (% of dietary protein)	4.13	5.11	6.04	6.91	7.76	8.69
Lysine	2.29	2.81	3.32	3.80	4.27	4.78
Arginine	3.15	3.17	3.19	3.15	3.16	3.18
Histidine	1.58	1.56	1.55	1.57	1.58	1.57
Isoleucine	3.07	3.06	3.10	3.04	3.07	3.08
Leucine	4.43	4.45	4.44	4.46	4.45	4.47
Methionine	2.25	2.26	2.24	2.28	2.27	2.26
Phenylalanine	2.73	2.75	2.74	2.76	2.74	2.77
Threonine	2.70	2.71	2.67	2.74	2.72	2.69
Valine	3.80	3.83	3.85	3.82	3.84	3.81
Total EAA	26.00	26.60	27.10	27.62	28.10	28.61
Non-essential amino acid (NEAA) profile						
Alanine	2.39	2.40	2.45	2.42	2.43	2.41
Aspartic acid	3.71	3.75	3.70	3.71	3.73	3.72
Cysteine	0.47	0.49	0.48	0.47	0.50	0.51
Glutamic acid	7.62	7.63	7.65	7.65	7.61	7.60
Glycine	5.24	4.69	4.23	3.69	3.17	2.68
Proline	2.74	2.75	2.79	2.77	2.78	2.76
Serine	2.12	2.11	2.08	2.09	2.10	2.13
Tyrosine	1.52	1.53	1.56	1.55	1.53	1.51
Total NEAA	25.81	25.35	24.94	24.35	23.85	23.32

^1^ Provided by Shandong Conqueren Marine Technology Co., Ltd., Weifang, China. ^2^ Amino acid mix contained (g per 100 g dry diet): arginine, 0.61; histidine, 0.60; methionine, 1.37; isoleucine, 0.75; leucine, 0.90; phenylalanine, 0.49; threonine, 0.89; tryptophan, 0.24; valine, 1.69. ^3^ Composition (IU or g/kg vitamin premix): retinal palmitate, 10,000 IU; cholecalciferol, 4000 IU; DL–α–tocopherol acetate, 75.0 g; menadione, 22.0 g; thiamin–HCl, 40.0 g; riboflavin, 30.0 g; D–calcium pantothenate, 150.0 g; pyridoxine–HCl, 20.0 g; meso–inositol, 300.0 g; D–biotin, 1.0 g; folic acid, 15.0 g; niacin, 200.0 g; cyanocobalamin, 0.3 g. ^4^ Composition (g/kg mineral premix): AlK(SO_4_)_2_·12H_2_O, 124.0; CaCl_2_, 17,880.0; CoC_l2_·6H_2_O, 49.0; FeSO_4_·7H_2_O, 707.0; KCl, 1192.0; KI, 5.0; MgSO_4_·7H_2_O, 4317.0; MnSO_4_·4H_2_O, 31.0; NaCl, 4934.0; Na_2_SeO_3_·H_2_O, 3.0; ZnSO_4_·7H_2_O, 177.0; Ca(H_2_PO_4_)_2_·H_2_O, 12,457.0; KH_2_PO_4_, 9930.0.

**Table 3:** Essential amino acid (EAA) composition of the ingredients in the experimental diets (% dry matter) ^1^.

EAAs	15% Fish Meal	10% Poultry By-Product Meal	15% Soy Protein Concentrate	10% Corn Gluten Meal	10% Wheat Gluten Meal	5% Beer Yeast	55.78% Coho Salmon Eyed Egg Protein	Supplied EAA
Arginine	0.71	0.38	0.81	0.22	0.33	0.12	3.18	0.61
Histidine	0.26	0.10	0.25	0.13	0.19	0.06	1.59	0.60
Methionine	0.35	0.09	0.14	0.15	0.14	0.04	2.28	1.37
Phenylalanine	0.54	0.19	0.53	0.46	0.35	0.22	2.78	0.49
Leucine	0.76	0.32	0.84	1.03	0.33	0.25	4.43	0.90
Isoleucine	0.50	0.16	0.48	0.43	0.60	0.15	3.07	0.75
Threonine	0.53	0.20	0.43	0.30	0.23	0.12	2.70	0.89
Valine	0.53	0.21	0.51	0.33	0.37	0.18	3.82	1.69
Lysine	0.90	0.28	0.56	0.12	0.16	0.18	4.06	Variable

^1^ Tryptophan was not determined due to acid hydrolysis.

**Table 4:** Survival and growth performance of coho salmon (*Oncorhynchus kisutch*) alevins that were fed the experimental diets with graded levels of L-lysine for 12 weeks ^1^.

Diets Groups	Analyzed Dietary L-Lysine Level (% of Diet)					
	2.29	2.81	3.32	3.80	4.27	4.78
Survival (%)	94.67 ± 1.53	96.33 ± 1.15	98.00 ± 1.00	97.00 ± 1.00	96.33 ± 0.58	95.67 ± 1.53
Initial body weight (g)	0.30 ± 0.01	0.30 ± 0.01	0.30 ± 0.01	0.30 ± 0.01	0.30 ± 0.01	0.30 ± 0.01
Final body weight (g)	4.39 ± 0.05 ^c^	4.70 ± 0.06 ^b^	4.94 ± 0.05 ^a^	5.09 ± 0.05 ^a^	4.86 ± 0.04 ^ab^	4.76 ± 0.02 ^b^
SGR (%/d)	3.19 ± 0.01 ^c^	3.27 ± 0.02 ^b^	3.33 ± 0.01 ^a^	3.37 ± 0.01 ^a^	3.31 ± 0.01 ^ab^	3.28 ± 0.01 ^b^
Feed intake (g)	4.89 ± 0.03 ^a^	4.84 ± 0.04 ^a^	4.69 ± 0.03 ^b^	4.73 ± 0.03 ^b^	4.79 ± 0.03 ^ab^	4.88 ± 0.02 ^a^
FCR	1.20 ± 0.02 ^a^	1.11 ± 0.02 ^b^	1.01 ± 0.02 ^c^	0.99 ± 0.01 ^c^	1.05 ± 0.01 ^bc^	1.09 ± 0.02 ^b^
PER (%)	1.52 ± 0.02 ^c^	1.65 ± 0.03 ^b^	1.80 ± 0.03 ^a^	1.84 ± 0.02 ^a^	1.73 ± 0.02 ^ab^	1.66 ± 0.03 ^b^
BPD (%)	20.22 ± 0.16 ^c^	22.18 ± 0.33 ^b^	25.15 ± 0.38 ^a^	26.10 ± 0.46 ^a^	24.85 ± 0.41 ^ab^	24.05 ± 0.39 ^b^
CF (%)	1.25 ± 0.07	1.34 ± 0.11	1.23 ± 0.13	1.30 ± 0.09	1.36 ± 0.16	1.21 ± 0.20
HSI (%)	1.69 ± 0.07	1.71 ± 0.06	1.74 ± 0.09	1.64 ± 0.12	1.62 ± 0.14	1.56 ± 0.17
VSI (%)	7.51 ± 0.10	7.35 ± 0.09	7.47 ± 0.07	7.42 ± 0.11	7.26 ± 0.06	7.32 ± 0.08

^1^ Means ± S.D. (*n* = 3) with the same superscript letter in the same row are not significantly different (*p* > 0.05).

**Table 5:** Whole-body proximate composition (% dry matter) of coho salmon (*Oncorhynchus kisutch*) alevins at the start of feeding trial and of alevins that were fed the experimental diets with graded levels of L-lysine for 12 weeks ^1^.

Dietary Groups (L-Lysine Level, % of Diet)	Moisture	Crude Protein	Crude Lipid	Ash
		The alevins at the start of feeding trial		
	77.85 ± 0.39	12.55 ± 0.22	6.01 ± 0.13	4.41 ± 0.18
		The alevins at the end of feeding trial		
2.29	78.02 ± 0.21	13.25 ± 0.16 ^c^	5.57 ± 0.20 ^a^	4.07 ± 0.29
2.81	77.56 ± 0.15	13.44 ± 0.12 ^c^	5.40 ± 0.09 ^a^	4.12 ± 0.31
3.32	77.70 ± 0.23	13.91 ± 0.09 ^b^	5.35 ± 0.11 ^ab^	4.18 ± 0.16
3.80	77.48 ± 0.25	14.11 ± 0.07 ^ab^	5.23 ± 0.14 ^b^	4.09 ± 0.25
4.27	77.63 ± 0.17	14.26 ± 0.08 ^ab^	5.17 ± 0.16 ^bc^	4.20 ± 0.33
4.78	77.56 ± 0.32	14.33 ± 0.11 ^a^	4.96 ± 0.17 ^c^	3.98 ± 0.22

^1^ Means ± S.D. (*n* = 3) with the same superscript letter in the same column are not significantly different (*p* > 0.05).

**Table 6:** Essential amino acid (EAA) profile (% dry matter) in the whole-body of coho salmon (*Oncorhynchus kisutch*) alevins that were fed diets with graded levels of L-lysine for 12 weeks ^1^.

Amino Acids	Analyzed Dietary L-Lysine Level (% of Diet)					
	2.29	2.81	3.32	3.80	4.27	4.78
Essential amino acid (EAA) profile						
Arginine	3.03 ± 0.04 ^b^	3.16 ± 0.03 ^b^	3.28 ± 0.03 ^a^	3.33 ± 0.02 ^a^	3.39 ± 0.03 ^a^	3.36 ± 0.03 ^a^
Histidine	1.05 ± 0.02	1.01 ± 0.03	1.11 ± 0.03	1.14 ± 0.02	1.08 ± 0.02	1.16 ± 0.03
Isoleucine	2.82 ± 0.03	2.73 ± 0.03	2.66 ± 0.02	2.77 ± 0.03	2.69 ± 0.03	2.64 ± 0.02
Leucine	4.03 ± 0.03	4.09 ± 0.03	4.14 ± 0.03	4.17 ± 0.03	4.11 ± 0.03	4.06 ± 0.03
Lysine	3.94 ± 0.03 ^b^	4.02 ± 0.03 ^b^	4.15 ± 0.03 ^a^	4.22 ± 0.04 ^a^	4.25 ± 0.04 ^a^	4.19 ± 0.03 ^a^
Methionine	2.15 ± 0.02	2.03 ± 0.03	2.07 ± 0.03	2.13 ± 0.03	2.23 ± 0.03	2.17 ± 0.03
Phenylalanine	2.56 ± 0.04	2.65 ± 0.04	2.73 ± 0.03	2.72 ± 0.03	2.62 ± 0.03	2.69 ± 0.03
Threonine	2.35 ± 0.03	2.26 ± 0.03	2.29 ± 0.03	2.19 ± 0.03	2.37 ± 0.03	2.15 ± 0.03
Valine	3.95 ± 0.04 ^b^	4.10 ± 0.03 ^b^	4.22 ± 0.03 ^a^	4.30 ± 0.05 ^a^	4.33 ± 0.04 ^a^	4.41 ± 0.04 ^a^
Total EAA^2^	25.88 ± 0.07 ^b^	26.05 ± 0.06 ^b^	26.65 ± 0.05 ^a^	26.97 ± 0.08 ^a^	27.07 ± 0.06 ^a^	26.83 ± 0.07 ^a^
Non-essential amino acid (NEAA) profile						
Alanine	3.50 ± 0.03	3.44 ± 0.03	3.53 ± 0.04	3.57 ± 0.04	3.46 ± 0.03	3.51 ± 0.03
Aspartic acid	4.45 ± 0.03	4.43 ± 0.03	4.51 ± 0.04	4.48 ± 0.03	4.46 ± 0.03	4.47 ± 0.03
Cystine	0.53 ± 0.02	0.51 ± 0.03	0.55 ± 0.03	0.54 ± 0.03	0.56 ± 0.02	0.52 ± 0.03
Glutamic acid	7.22 ± 0.04	7.27 ± 0.04	7.33 ± 0.05	7.35 ± 0.04	7.19 ± 0.04	7.26 ± 0.04
Glycine	3.72 ± 0.03 ^a^	3.67 ± 0.03 ^a^	3.56 ± 0.02 ^b^	3.49 ± 0.03 ^b^	3.38 ± 0.03 ^bc^	3.31 ± 0.03 ^c^
Proline	2.67 ± 0.02	2.66 ± 0.02	2.62 ± 0.03	2.69 ± 0.03	2.76 ± 0.03	2.72 ± 0.03
Serine	2.23 ± 0.03	2.29 ± 0.03	2.25 ± 0.03	2.31 ± 0.03	2.26 ± 0.03	2.23 ± 0.03
Tyrosine	2.39 ± 0.03	2.35 ± 0.03	2.34 ± 0.03	2.41 ± 0.03	2.37 ± 0.03	2.45 ± 0.03
Total NEAA	26.71 ± 0.05	26.62 ± 0.05	26.69 ± 0.07	26.84 ± 0.06	26.44 ± 0.05	26.47 ± 0.06

^1^ Means ± S.D (standard deviation, *n* = 3) with the same superscript letter in the same row are not significantly different (*p* > 0.05). ^2^ Tryptophan was not determined due to acid hydrolysis.

## Data Availability

The data presented in this study are available on request from the corresponding author. The data are not publicly available due to the confidentiality of the research projects.
